# Intraperitoneal *versus* intravenous administration of Flamma®-conjugated PEG-alendronate-coated upconversion nanoparticles in a mouse pancreatic cancer model†

**DOI:** 10.1039/d4na00764f

**Published:** 2024-10-25

**Authors:** Taras Vasylyshyn, Vitalii Patsula, David Větvička, Oleksandr Shapoval, Jan Pankrác, Martina Kabešová, Jiří Beneš, Daniel Horák

**Affiliations:** a Institute of Macromolecular Chemistry, Czech Academy of Sciences Heyrovského Nám. 2 162 00 Prague 6 Czech Republic horak@imc.cas.cz; b Institute of Biophysics and Informatics, First Faculty of Medicine, Charles University Salmovská 1 120 00 Prague 2 Czech Republic; c Center for Advanced Preclinical Imaging, First Faculty of Medicine, Charles University Salmovská 3 120 00 Prague 2 Czech Republic

## Abstract

Pancreatic cancer is one of the most common forms of malignant disease with a poor survival prognosis. Currently, nanomedicine holds great promise for targeted diagnosis and treatment of this cancer, which also reduces toxic side effects. In this work, we prepared PEG-coated monodisperse upconversion nanoparticles (UCNPs) with a conjugated Flamma® fluorescent dye for imaging and detection of particle distribution *in vivo*. We performed a thorough physicochemical characterization of the particles and determined their colloidal and chemical stability in several aqueous media such as water, PBS, Dulbecco's modified Eagle's medium and artificial lysosomal fluid. Luminescence resonance energy transfer from the emission of UCNPs as a donor to the Flamma® as an acceptor was confirmed. Intraperitoneal *versus* intravenous administration was then compared in terms of biodistribution of particles in various organs in the orthotopic mice pancreatic cancer model. The intraperitoneal route was preferred over the intravenous one, because it significantly increased the accumulation of particles in the tumor tissue. These new UCNP@Ale-PEG-Flamma® nanoparticles are thus promising for new treatment avenues for pancreatic cancer.

## Abstract 2

New PEG-coated upconversion nanoparticles conjugated with Flamma® dye demonstrated superior imaging and targeting of pancreatic tumor-bearing mice after intraperitoneal administration.

## Graphical abstract

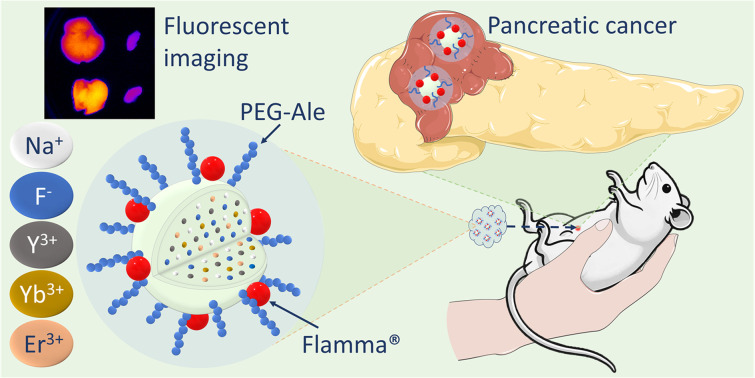

## Introduction

Pancreatic cancer is a highly resistant disease that is becoming increasingly widespread and difficult to treat.^1^ Progress in this area is made by new nanotechnologies including lanthanide-doped upconversion nanoparticles (UCNPs). They are attracting growing interest especially in biomedical imaging, cancer treatment, photodynamic cell therapy, biosensing (*e.g.* nanothermometry or upconversion-linked immunosorbent assays), optogenetics, photoswitching and lasing^2^ due to their unique optical properties converting near-infrared (NIR) light into UV-vis emission. These features include large anti-Stokes shifts, intense and stable photoluminescence detectable for single particles, sharp emission bands, tunable emissions, and absence of photoblinking and photobleaching.^3^ In addition, the use of NIR light allows deep penetration into tissues, thus minimizing unwanted cell damage and autofluorescence.

A number of methods have been described to synthesize UCNPs with controlled morphology (shape, size and particle size distribution) and crystallinity, such as hydro(solvo)thermal synthesis, coprecipitation, thermal decomposition and their modifications.^4^ During syntheses, particles are formed by gradual nucleation, crystal growth and Ostwald ripening. These processes are carried out in organic solvents, typically in the presence of oleic acid, resulting in the formation of hydrophobic particles which then need to be transferred by various procedures to the aqueous environment required by biological applications. Therefore, oleic acid must be replaced by biocompatible coatings that contain various functional groups (amino, carboxyl, sulfhydryl, hydroxyl, phosphonate, *etc.*) that bind to the surface of UCNPs and additionally allow conjugation of target biomolecules. These coatings are very diverse, ranging from silica to poly(acrylic acid), poly(ethylene glycol) (PEG) derivatives, polyethyleneimine, polyvinylpyrrolidone, poly(*N*,*N*-dimethylacrylamide), poly(maleic anhydride-*alt*-1-octadecene), poly(4-styrenesulfonic acid-*co*-maleic anhydride), chitosan, dextran, phospholipids, *etc.*, and provide steric or electrostatic stabilization.^5–7^ In particular, PEG has proven to be a good choice here because it is not only biocompatible and hydrophilic, but also avoids clearance by the reticuloendothelial system and ensures long circulation in the blood.^8^ Preferably, PEG is functionalized with aminobisphosphonates such as alendronate or neridronate,^9^ which are widely used for osteoporosis treatment and in bone tissue engineering. The advantage of the bisphosphonate complexing group is that it firmly anchors the PEG to the UCNP surface and ensures their colloidal stability and biocompatibility.

To improve bioimaging, it is convenient to combine surface-modified UCNPs with fluorescent dyes, the physicochemical properties of which affect the results of biodistribution studies.^10^ These dyes are exemplified by rhodamine, cyanine, Nile red, *etc.*, which improve the detection of certain compounds such as metal ions, hydrazine, cyanide, amino acids, enzymes, DNA, or mycotoxin.^11^ Another example of an interesting NIR fluorescent imaging dye is Flamma® Fluor 749 (Flamma® for short) containing various reactive functional groups showing specific reactivity towards primary amines such as lysine. Flamma® covers the entire spectral range from UV to NIR with excellent fluorescence performance. It is characterized by strong absorption, high quantum yield of fluorescence and good photostability. The high fluorescence activity and stability of Flamma® dyes are retained even after conjugation with biomolecules, enabling highly sensitive multimodal imaging and detection of low-abundance biological structures. For example, intravenously injected Flamma® was used to localize docetaxel-loaded poly(ethylene oxide)-*block*-poly(propylene oxide)-*block*-poly(ethylene oxide) nanoparticles in tumor-bearing mice.^12^

Anticancer drug delivery often uses intravenous injection as the first choice of administration. In some scenarios, this is actually the best option. The problem is that most of the earlier animal studies were performed on subcutaneously growing tumor models, which are more artificial than the currently preferred orthotopic models. The importance of utilizing orthotopic models is especially true in the case of pancreatic cancer. Orthotopically growing pancreatic tumor models mimic clinical pathophysiology much better than conventional models. Subcutaneously growing pancreatic tumors are much more vascularized than their orthotopic counterparts and this increased vascularization enables efficient drug accumulation based on the enhanced permeation and retention effect (EPR).^13^ In general, however, pancreatic tumors are poorly vascularized with very dense stroma,^14^ which plays against the EPR effect. Some studies suggest that in the case of intraperitoneal tumors, intraperitoneal drug delivery systems are superior to more common intravenous administration.^15,16^ Intraperitoneal injection may enhance retention of nanomedicines in the peritoneal cavity, providing more opportunities for direct contact of tumors with high therapeutic concentrations. It is believed that intraperitoneally injected particles preferentially localize in peritoneal tumors rather than in normal organs due to differences in the surface mesothelium.^17^ The abdominal organs are covered by intact mesothelium and submesothelial fibrous connective tissue, which act as barriers against adhesion and entry of nanoparticles. In contrast, peritoneal tumors typically lack these protective surface coverings due to disruption of mesothelial cells during tumor progression, resulting from interactions between tumor cells and mesothelial peritoneum.

In this report, Flamma® Fluor 749 fluorescent dye was covalently attached to PEG-Ale as a stabilizing coating of UCNPs *via* an amide bond. These surface-modified UCNPs were injected into mice bearing an orthotopically growing Panc02 murine pancreatic tumor model and intravenous *vs.* intraperitoneal administration was compared in terms of particle accumulation in the tumor.

## Experimental

### Materials

The sodium salt of 4-amino-1-hydroxy-1-phosphonobutyl phosphonic acid trihydrate (alendronate; Ale) was purchased from TCI (Tokyo, Japan). Dichloromethane (DCM; 99.9%), NaOH (98.6%), HCl (35%), and salts (Na_2_HPO_4_·12 H_2_O and KH_2_PO_4_) used for the preparation of 0.5 M phosphate buffer (PB) were obtained from Lach-Ner (Neratovice, Czech Republic). α-*Tert*-butyloxycarbonylamino-ω-carboxy succinimidyl ester poly(ethylene glycol) (Boc-NH-PEG-NHS; *M*_w_ = 5000 g mol^−1^) was purchased from Rapp Polymere (Tübingen, Germany). Flamma® Fluor 749 sulfo-NHS ester (Flamma®) was from BioActs (Incheon, Korea). Dulbecco's modified Eagle's medium (DMEM), phosphate-buffered saline (PBS; pH 7.4) and fetal bovine serum (FBS) were purchased from Sigma-Aldrich (St. Louis, MO, USA). Artificial lysosomal fluid (ALF; pH 4.5) was prepared as described elsewhere.^18^ Artificial peritoneal fluid (APF) was prepared by modifying a previously published method.^19,20^ Also, NaYF_4_:Yb,Er nanoparticles (UCNPs) were prepared according to our earlier report.^21^ Ultrapure Q-water ultrafiltered using a Milli-Q Gradient A10 system (Millipore; Molsheim, France) was used in all experiments.

### Synthesis of PEG-alendronate (PEG-Ale)

**Fig. 1:**
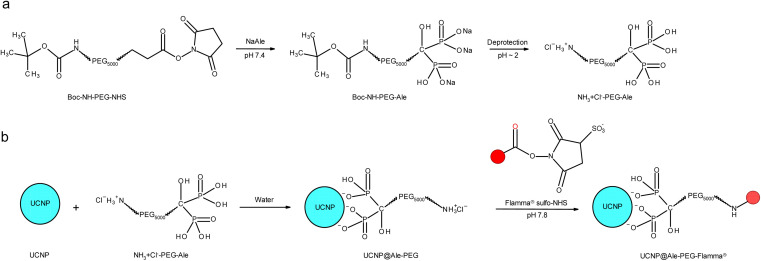
(a) Modification of PEG with Ale followed by deprotection and (b) modification of UCNPs with PEG-Ale and Flamma® (red sphere).

PEG-Ale was synthesized according to a previously published procedure (Fig. 1a).^22^ Briefly, alendronate (0.14 g; 0.56 mmol) was dissolved in 0.05 M PB (4 ml; pH 7.4); the pH was adjusted to 7.4 by adding 4 M aqueous NaOH and the solution was cooled to 0 °C. Boc-NH-PEG-NHS (0.3 g) was added and the reaction mixture was stirred at 0 °C for 6 h and at room temperature (RT) for 16 h. The mixture was acidified to pH 2 with 4 M HCl to release the Boc-protected amino groups, and PEG-Ale was separated by centrifugation after DCM extraction (3 × 8 ml). Finally, DCM was removed on a rotary evaporator at 30 °C and the resulting product was vacuum-dried at 60 °C over phosphorus pentoxide.

### Preparation of UCNP@Ale-PEG-Flamma® nanoparticles

Synthesis of UCNP@Ale-PEG-Flamma® nanoparticles is schematically shown in Fig. 1b. Briefly, PEG-Ale (70 mg) was added to an aqueous dispersion (1.5 ml) of neat UCNPs (40 mg), and the mixture was stirred at 80 °C for 12 h and stored in a refrigerator for 12 h. The resulting UCNP@Ale-PEG nanoparticles were separated by centrifugation (14 100 rcf) for 30 min, washed in water (3 times) and redispersed in water. The pH of the dispersion was adjusted to 7.8 using carbonate buffer, and an aqueous solution (0.5 ml) of Flamma® (2 mg; ESI, Fig. S1†) was added and the mixture was magnetically stirred at RT for 24 h in the dark. Excess Flamma® was removed by washing the particles with water by centrifugation (14 100 rcf) for 10 min three times.

### Physicochemical characterization of particles

Transmission electron micrographs, from which the number-average diameter *D*_n_ and the dispersity *Đ* were evaluated from at least 300 particles, were taken with a Tecnai G2 Spirit Twin 12 transmission electron microscope (TEM; FEI; Brno, Czech Republic). The weight-average diameter (*D*_w_ = Σ*N*_i_·*D*_i_^4^/Σ*N*_i_·*D*_i_^3^), number-average diameter (*D*_n_ = Σ*N*_i_·*D*_i_/Σ*N*_i_), and dispersity (*Đ* = *D*_w_/*D*_n_) were calculated using Atlas software (Tescan Digital Microscopy Imaging; Brno, Czech Republic). Alternatively, a dispersion of particles in hexane (2 μl) was dropped onto a freshly glow-discharged CF400-Cu carbon grid and the solvent evaporated in air; micrographs were taken in an HR-TEM JEM-2100Plus (JEOL; Tokyo, Japan) at 200 kV and magnifications of 50 000× and 300 000× using a TemCam-XF416 4K CMOS camera (TVIPS; Gauting, Germany). Dynamic light scattering (DLS) was measured using a ZSU 5700 Zetasizer Ultra instrument (Malvern Instruments; Malvern, UK) to determine the hydrodynamic diameter *D*_h_, polydispersity PD and *ζ*-potential. Infrared spectra were measured on a 100T FTIR spectrometer (PerkinElmer; Waltham, MA, USA) using a Specac MKII Golden Gate single attenuated total reflection (ATR) system. Thermogravimetric analysis (TGA) was performed in an oxygen atmosphere in a temperature range of 30–850 °C at a heating rate of 10 °C min^−1^ using a PerkinElmer TGA 7 analyzer (Norwalk, CT, USA). Absorption spectra in the ultraviolet and visible region (UV-vis) were measured using a Specord 250 Plus UV-vis spectrophotometer (Analytik Jena; Jena, Germany). Excitation and emission luminescence spectra were measured in a Hellma 114F-QS cuvette (10 × 4 mm path length; Sigma-Aldrich) at RT using an FS5 spectrofluorometer (Edinburgh Instruments; Edinburgh, UK) equipped with a 150 W continuous xenon lamp and an infrared diode laser with a wavelength of 980 nm and a nominal power of 2 W (MDL-III-980; beam size 5 × 8 mm^2^).

### Colloidal and chemical stability of UCNP@Ale-PEG nanoparticles

Dispersions of neat UCNPs and UCNP@Ale-PEG particles in water, DMEM with 10% FBS, ALF, and 0.01 M PBS (pH 7.4; each 1 mg ml^−1^) were shaken (300 rpm) in 2 ml plastic vials sealed with rubber septa at 37 °C for the given time. The dispersions were then redispersed using an ultrasonic bath for 30 s, and colloidal stability was determined by DLS measurement of hydrodynamic size (*D*_h_) and *ζ*-potential at 25 °C. The UCNPs were then separated from the dispersions by centrifugation (14 130 rcf) for 35 min and the supernatants were filtered (MWCO = 30 kg mol^−1^) to remove residual particles. The amount of released F^−^ ions, defined as the molar percentage of F^−^ (*X*_F_) relative to the fluorine content of the nanoparticles, was measured with a combined fluoride electrode (Thermo Fisher Scientific; Waltham, MA, USA). In addition, the colloidal stability of UCNP@Ale-PEG-Flamma® particles in FBS and APF for 24 h was investigated according to the above procedure to model the *in vivo* conditions.

### Cell line and laboratory mice

The murine pancreatic adenocarcinoma cell line Panc02 was obtained from Dr Smolková, Institute of Physiology, Czech Academy of Sciences. Cells were grown in RPMI 1640 medium (Gibco; Waltham, MA, USA) supplemented with 10% FBS, 1% sodium pyruvate, 1% penicillin/streptomycin, 2 mM glutamine and glucose (4.5 g l^−1^) in a 5% CO_2_ atmosphere in a humidified incubator heated to 37 °C.

Syngeneic 10 weeks-old female C57Bl/6 mice (AnLab; Prague, Czech Republic) were housed in individually ventilated cages to minimalize the risk of potential infection, in 12 h light/dark cycles. Autoclaved water and irradiated Ssniff diet (Ssniff Spezialdiaeten; Soest, Germany) were freely accessible *ad libitum*. The reported *in vivo* experiments were approved by the Ethics Committee of the First Faculty of Medicine of Charles University and the Ministry of Education, Youth and Sports of the Czech Republic. The study followed the guidelines of Decree 419/2012 on the protection of experimental animals and Act No. 246/1992 Sb. on the protection of animals against cruelty, both in compliance with the legislation of the European Union.

### Design of an orthotopic pancreatic model

Subconfluently growing Panc02 cells were washed with preheated PBS and incubated for 10 min in trypsin-ethylenediaminetetraacetic acid solution. The trypsin-based detachment mechanism was blocked after 10 min by the addition of FBS (5 ml). Harvested cells were washed twice in RPMI 1640 medium without FBS, and cell concentration was counted using an automated Luna cell counter (Logos Biosystems, distributed by KRD; Prague, Czech Republic). Cells were further diluted in RMPI 1640 medium without FBS to the desired concentration of 2 × 10^7^ Panc02 cells.

A total of 10 female C57Bl/6 mice with pre-shaved abdomen were anesthetized with an intraperitoneal injection of ketamine–xylazine (50 mg ml^−1^ ketamine; 5 mg ml^−1^ combined xylazine solution) at a dose of 0.05 ml for ∼25 g mice, the abdomen was disinfected and a 1–1.5 cm long incision was made in the left upper site. The pancreatic tail was placed under the spleen and gently pulled through the incision. A total of 50 μl of an ice-cold mixture of Matrigel and 5 × 10^5^ Panc02 cells was injected into the pancreatic tail using a precooled syringe with a 27-gauge needle. The exposed pancreas was gently returned to the peritoneum and the incision was carefully closed with Tervalon braided HR18/1 1.5EP surgical suture (Chirana T. Injecta; Prague, Czech Republic). Wound healing and any signs of pain were monitored daily for 5 days; no discomfort or wound inflammation was noted. In accordance with our pilot experiments, visible tumors appeared 7–8 days after injection.

### *In vivo* biodistribution of UCNPs

On the eighth day after surgery and tumor cell inoculation, mice were randomly divided into two groups of 5 mice each. Each group was injected either intraperitoneally or intravenously (tail vein) with 100 μl of UCNP@Ale-PEG-Flamma® particle (0.15 mg) dispersion. After another 24 h, mice were euthanized and tumors and selected tissues (liver, kidney and muscle) were dissected. From each group (intraperitoneal or intravenous), 4 mice were selected for further analysis, excluding the smallest tumor from both groups. The collected tissues were placed on a Petri dish, with tissues from two animals per dish. The tissues were then imaged using an In-Vivo Xtreme™ optical imaging unit (Bruker; Billerica, MA, USA). The following settings were used for imaging Flamma® fluorescence: band filter 750 nm for excitation, band filter 830 nm for emission, aperture f/1.1, exposure time 60 s. Image processing and fluorescence quantification were performed in the open-source software platform Fiji.^23^

## Results and discussion

### Flamma®-conjugated PEG-Ale-coated UCNPs (UCNP@Ale-PEG-Flamma®)

NaYF_4_:Yb,Er nanoparticles (UCNPs) were obtained by oleic acid-stabilized thermal coprecipitation of lanthanide chlorides in octadec-1-ene at 300 °C. To render these hydrophobic particles useable for biomedical applications, it was necessary to achieve their colloidal stability in aqueous media. Therefore, the synthesized UCNPs were thoroughly washed with hexane, ethanol and water and subsequently coated with PEG-Ale containing the terminal amino group (Fig. 1). Afterward, Flamma® Fluor 749 sulfo-NHS ester reacted with amino groups of PEG-coated UCNPs. The advantage of sulfo-NHS activated Flamma® consists in its solubility in aqueous media, which is a prerequisite for the use of UCNPs in life science applications where the use of organic solvents such as dimethyl sulfoxide or *N*,*N*-dimethylformamide must be avoided. Moreover, Flamma® allows a fast and easy determination of particle distribution in organs using optical imaging due to its excellent fluorescence.

**Fig. 2:**
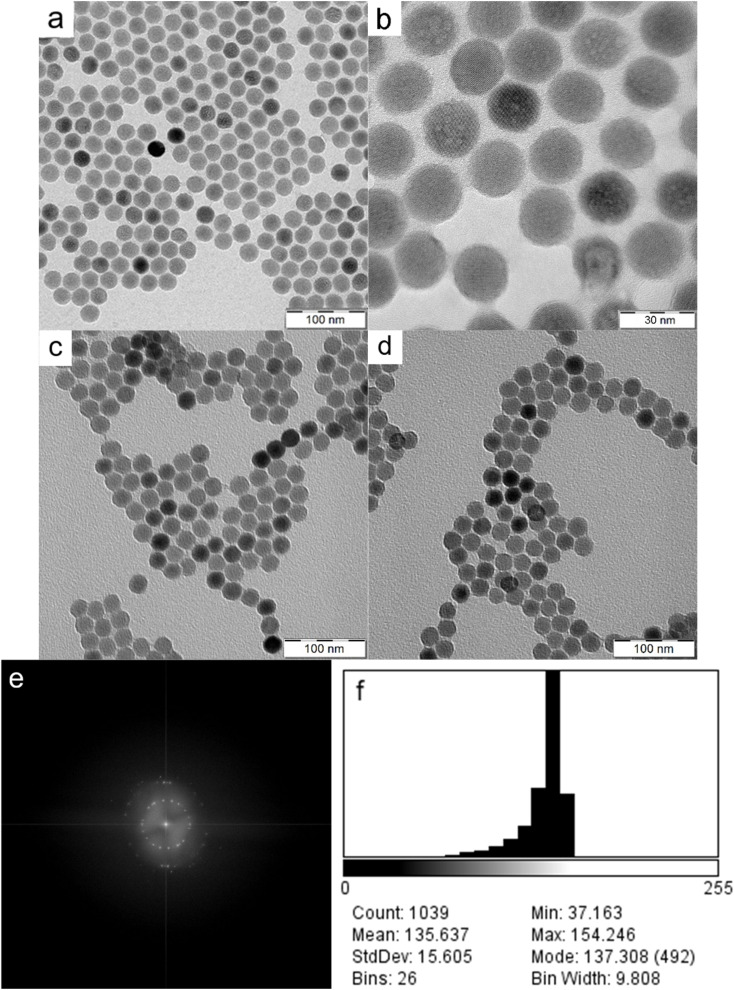
(a and b) HR-TEM and (c and d) TEM micrographs of (a and b) neat UCNPs, (c) UCNP@Ale-PEG and (d) UCNP@Ale-PEG-Flamma® nanoparticles. (e) FFT diffraction pattern and (f) electron density distribution (mean grey value) of UCNPs.

As shown by HR-TEM, the UCNPs were 22.9 ± 1.1 nm in diameter and invariably round-shaped (Fig. 2a and b). The particles became unstable after exposure to the condensed e-beam; note the blebs appearing on some UCNPs under 3 00 000× magnification (Fig. 2b). The fast Fourier transformation (FFT) diffraction images showed clearly discernible lattice fringes confirming the crystal structure of particles (Fig. 2e). Electron density distribution measurements showed slight heterogeneity within the population with a mean grey value of 136 ± 16 (range 0–255) and a negatively skewed distribution with a minor fraction of significantly darker particles (Fig. 2f). Similarly, the dry UCNP@Ale-PEG and UCNP@Ale-PEG-Flamma® particles had a spherical shape of 24 nm and were monodisperse (dispersity *Đ* = 1.01; Fig. 2c and d). It should be mentioned that particle uniformity is important for product quality, homogeneous luminescence and reproducibility of biological performance. TEM micrographs also showed that the polymer coating did not produce contrast, so only the lanthanide UCNPs were visible. Another important parameter characterizing the size of particles in water is their hydrodynamic diameter (*D*_h_) measured by DLS (Table 1). The *D*_h_ of neat UCNPs reached ∼100 nm, while UCNP@Ale-PEG particles were smaller (*D*_h_ = ∼90 nm), which was in agreement with our previous report.^24^ Not surprisingly, the *D*_h_ of neat particles in water was always larger than the *D*_n_ of dry particles according to TEM, which measures a number-average diameter, while the DLS provides the z-average-weighted light intensity. Moreover, a slight aggregation of particles in water cannot be ruled out. In the case of the UCNP@Ale-PEG-Flamma® particles, the *D*_h_ increased to 124 nm due to the formation of a solvation layer of counterions. The polydispersity PD measured by DLS oscillated between ∼0.15 and 0.20. While the *ζ*-potential of UCNPs amounting to 31 mV was highly positive due to the presence of positively charged Ln ions on the surface, the *ζ*-potential of UCNP@Ale-PEG was lower (18 mV) due to shielding by electroneutral PEG (Table 1). The *ζ*-potential of UCNP@Ale-PEG-Flamma® nanoparticles then reached negative values (−8 mV) due to the sulfonic acid groups of Flamma®.

**Table 1:** Characterization of nanoparticles^a^

Particles	*D*_n_ (nm)	*Đ*	*D*_h_ (nm)	PD	*ξ*-potential (mV)
UCNPs	24	1.01	105	0.14	31
UCNP@Ale-PEG			91	0.17	18
UCNP@Ale-PEG-Flamma®			124	0.21	−8

a *D*_n_ – number-average diameter (TEM), *Đ* – dispersity (TEM), *D*_h_ – hydrodynamic diameter (DLS), PD – polydispersity (DLS).

**Fig. 3:**
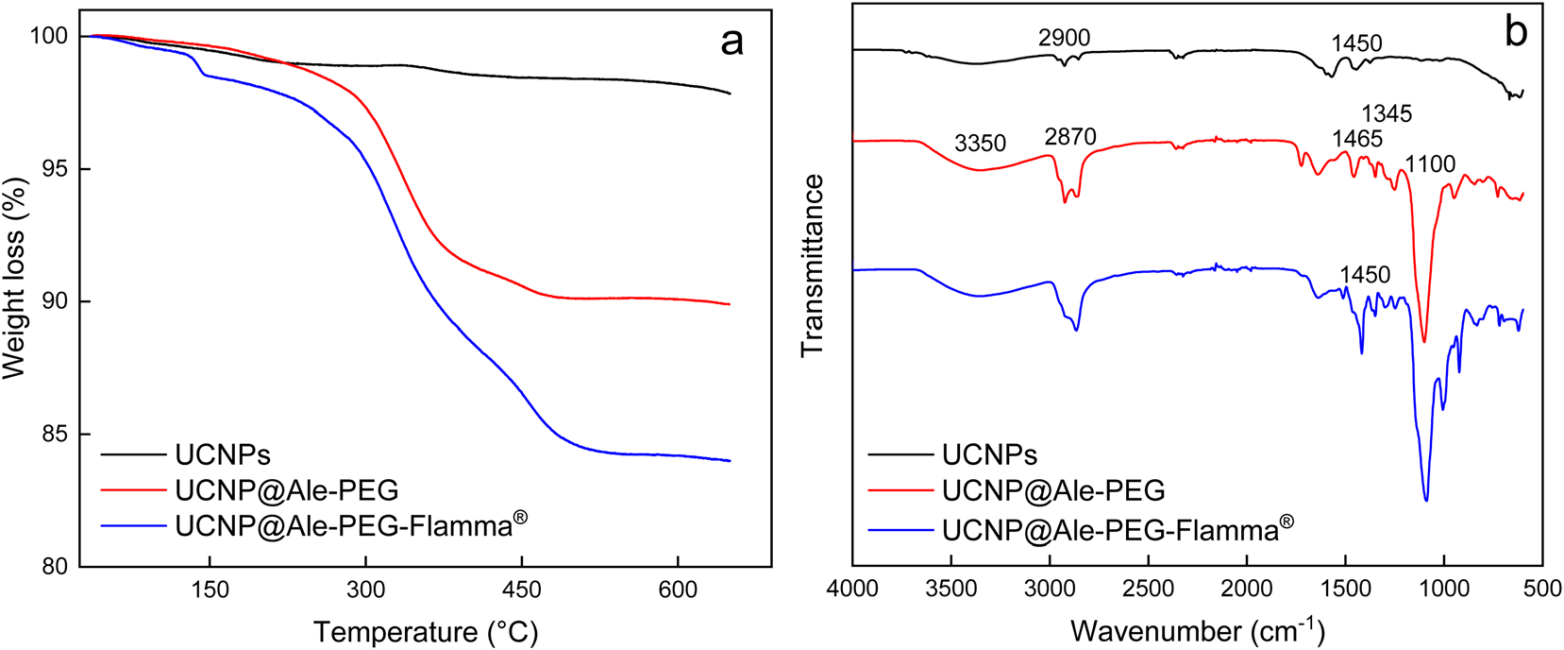
(a) TGA thermograms and (b) FTIR spectra of UCNPs, UCNP@Ale-PEG and UCNP@Ale-PEG-Flamma® particles.

TGA of neat UCNPs confirmed the presence of a small amount of oleic acid used as a stabilizer (Fig. 3a). Similarly, the FTIR spectrum of the particles showed small characteristic peaks of oleic acid at ∼2900 and ∼1450 cm^−1^ belonging to asymmetric and symmetric C–H stretching vibrations and CH_2_ bending vibrations, respectively (Fig. 3b). TGA thermograms of UCNP@Ale-PEG and UCNP@Ale-PEG-Flamma® particles confirmed successful modification by Ale-PEG and conjugation with Flamma®; the particles contained 8.2 wt% PEG and 6.5 wt% Flamma® (Fig. 3a).

**Fig. 4:**
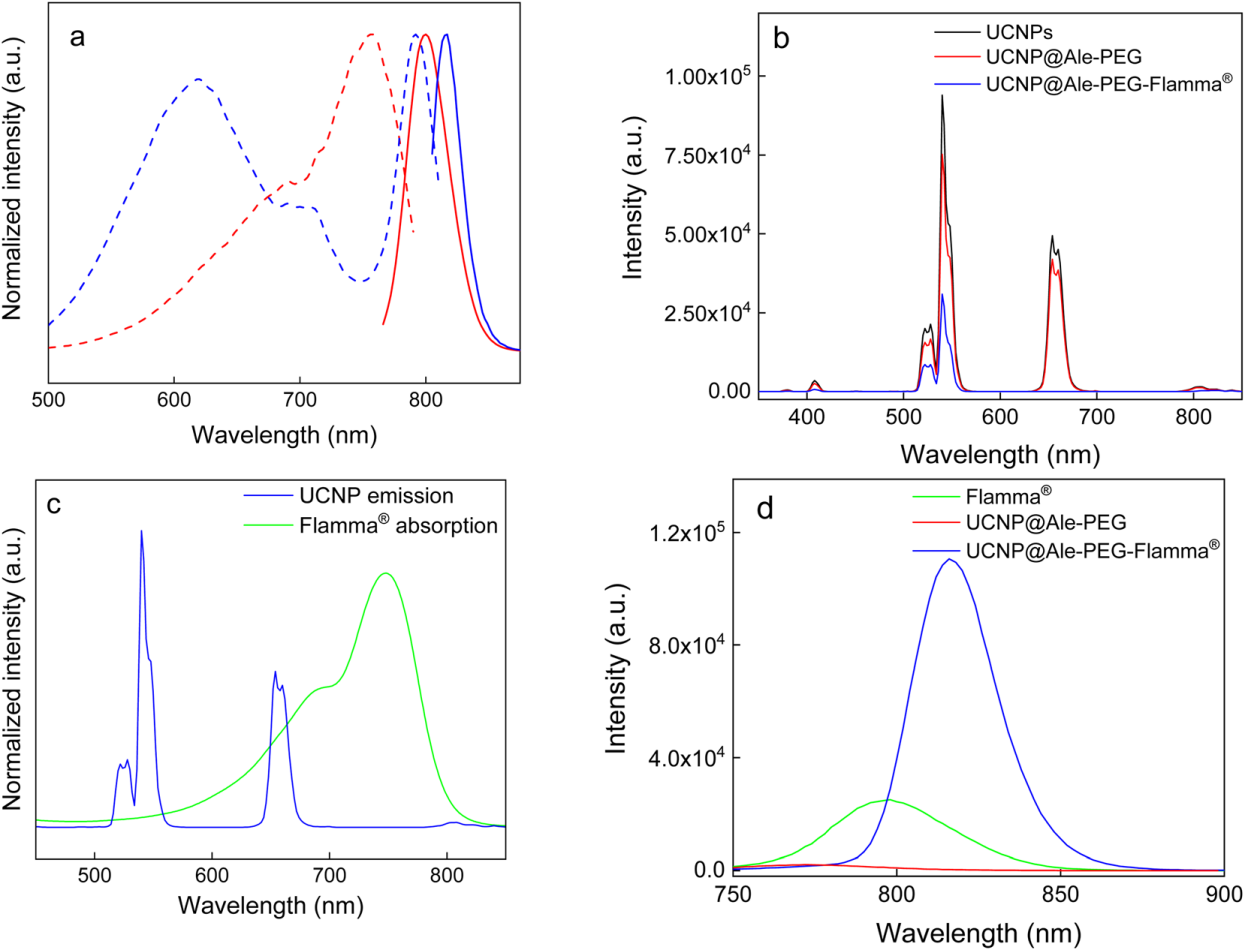
(a) Photoluminescence excitation (dash) and emission (solid) spectra of Flamma® (red; *λ*_ex_ 756 nm, *λ*_em_ 800 nm) and UCNP@Ale-PEG-Flamma® particles (blue; *λ*_ex_ 791 nm, *λ*_em_ 816 nm). (b) Upconversion emission spectra of UCNPs (black), UCNP@Ale-PEG (red), and UCNP@Ale-PEG-Flamma® particles (blue) in water (2 mg ml^−1^) at 980 nm excitation and a laser power density of 2.11 W cm^−2^. (c) UV-vis absorption of Flamma® and upconversion luminescence spectra of UCNPs. (d) Photoluminescence emission spectra of Flamma® (green), UCNP@Ale-PEG (red) and UCNP@Ale-PEG-Flamma® particles (blue) excited at 618 nm.

The FTIR spectrum of UCNP@Ale-PEG showed a broad peak at 3350 cm^−1^ corresponding to the stretching vibration of the O–H bond, peaks at 2870, 1465 and 1345 cm^−1^ assigned to the stretching of C–H and a band at ∼1100 cm^−1^ ascribed to the vibration of the C–O–C bond. In the spectrum of UCNP@Ale-PEG-Flamma® particles, the increase in peak intensity at ∼1450, 900 and 750–850 cm^−1^ can be explained by the presence of aromatic rings and C=C Flamma® bonds (Fig. 3b). The UV absorption spectrum of UCNP@Ale-PEG-Flamma® confirmed Flamma®-conjugation with UCNP@Ale-PEG particles (Fig. S2†). In contrast to the uncoated UCNPs and UCNP@Ale-PEG particles, the characteristic absorption peak of Flamma® at 747 nm was observed for UCNP@Ale-PEG-Flamma® particles. The conjugation of Flamma® to UCNP@Ale-PEG particles was also confirmed by the photoluminescence spectra of UCNP@Ale-PEG-Flamma® (Fig. 4a). While the maximum excitation and emission of free Flamma® occurred at 756 and 800 nm, respectively, after the excitation of UCNP@Ale-PEG-Flamma® particles at 791 nm, the intense emission was shifted to 816 nm due to the bound Flamma® (Fig. 4a). The shift of the excitation and emission peaks (by 35 and 16 nm, respectively) compared to the peak of free Flamma® was attributed to its conjugation with UCNP@Ale-PEG particles.

### Colloidal stability of UCNP@Ale-PEG nanoparticles

**Fig. 5:**
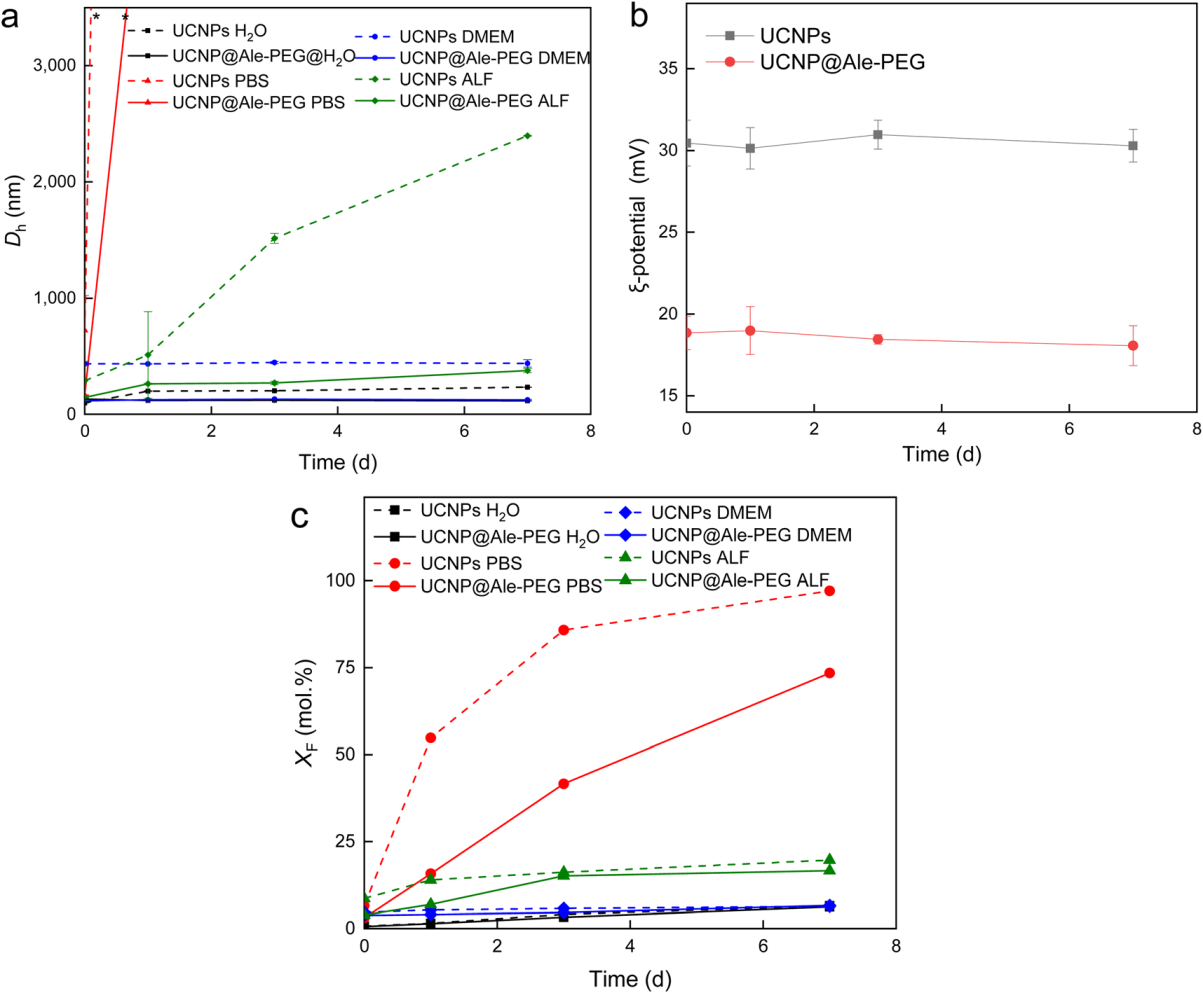
Time dependencies of (a) hydrodynamic diameter *D*_h_ in water, PBS, DMEM and ALF, (b) *ζ*-potential of UCNPs and UCNP@Ale-PEG nanoparticles in water and (c) F^−^ ion molar fraction (*X*_F_) in the supernatants after incubation of particles in water, PBS (pH 7.4), DMEM and ALF at 37 °C. * Beyond the scope of measurement.

The colloidal stability of UCNP@Ale-PEG particles was determined at 37 °C in water, DMEM, ALF and PBS, which are the media used in biological experiments. The hydrodynamic diameter of the particles (*D*_h_) and their *ζ*-potential were compared with the values for neat particles (Fig. 5a and b). The *ζ*-potential of particles in DMEM, ALF and PBS could not be determined due to interference of their components, such as amino acids, salts and vitamins, with the measurement. *D*_h_ of UCNP@Ale-PEG particles in water, DMEM and ALF remained almost unchanged over 7 days, thus the particles did not aggregate, indicating their good colloidal stability due to steric stabilization. However, in PBS, the *D*_h_ of UCNP@Ale-PEG particles increased already after one day of aging, similar to that of neat UCNPs (Fig. 5a). In the case of neat UCNP and UCNP@Ale-PEG particles in water, the *ζ*-potential did not change over 7 days (Fig. 5b). To model the behavior of UCNP@Ale-PEG-Flamma® particles under *in vivo* conditions, their colloidal stability was measured in FBS and APF. After 24 h, the *D*_h_ of UCNP@Ale-PEG-Flamma® particles did not change significantly in both FBS (*D*_h_ = 62–64 nm) and APF (*D*_h_ = 135–139 nm). This indicates good colloidal stability of the particles; however, the *ζ*-potential could not be measured due to interference of the components (proteins and salts) of both media with the evaluation of DLS.

### Chemical stability of UCNP@Ale-PEG nanoparticles

Previous studies have shown that, especially at elevated temperatures, lanthanide-based UCNPs are susceptible to dissolution in aqueous biological media, which can reduce the luminescence intensity of particles and induce cell death.^25,26^ In this work, the chemical stability of neat UCNPs and UCNP@Ale-PEG particles in selected model media such as water, PBS, DMEM and ALF was evaluated at 37 °C, which simulates the human body temperature (Fig. 5c). Similar to previous works,^27,28^ the particles showed only low solubility in water and relatively high solubility in PBS, which can be explained by the enhanced hydrolysis of the particles caused by the complexation of PBS phosphate ions with the lanthanides of UCNPs.^29^ In water and DMEM, the UCNP@Ale-PEG particles were chemically stable (*X*_F_ = 6.4 and 6.5 mol%), while in ALF and PBS the stability was only slightly better (*X*_F_ = 16.5 and 73.1 mol%, respectively) compared to that of the neat particles (*X*_F_ = 19.6 and 97.5 mol%, respectively) due to the phosphonate groups of alendronate binding to the surface of UCNPs and partially hindering thus the diffusion of phosphates to the particle surface. The protective effect of amino-terminated PEG-Ale in preventing particle degradation was similar to that of poly(*N*,*N*-dimethylacrylamide-*co*-2-aminoethylacrylamide) and PEG.^27^

### Photoluminescence properties of UCNP@Ale-PEG-Flamma® nanoparticles

The upconversion luminescence emission of neat UCNPs and UCNP@Ale-PEG particles was measured under NIR excitation at a wavelength of 980 nm and a laser power density of 2.11 W cm^−2^ (Fig. 4b). Bands at 408 nm (^2^H_9/2_ → ^4^I_15/2_), 525 nm (^2^H_11/2_ → ^4^I_15/2_), 540 nm (^4^S_3/2_ → ^4^I_15/2_), 654 nm (^4^F_9/2_ → ^4^I_15/2_) and 807 nm (^4^I_9/2_ → ^4^I_15/2_), which corresponded to the characteristic Er^3+^ emission transitions, were observed in the spectra. The modification of UCNPs with PEG-Ale slightly reduced the intensity of the upconversion emission. Conjugation of Flamma® with UCNP@Ale-PEG particles led to ∼3-fold lower upconversion intensity at 408, 525 and 540 nm and a significant quenching of luminescence (1000-fold) at 654 nm compared to UCNP@Ale-PEG particles (Fig. 4b). This was evident in the energy transfer at 654 nm (^4^F_9/2_ → ^4^I_15/2_) from UCNPs to Flamma® due to the spectral overlap of the upconversion emission of the particles and the Flamma® absorption (Fig. 4c). Such spectral overlap is a prerequisite for efficient energy transfer, provided that the distance between the interacting dye molecule and the particle surface is sufficiently small.^30^ Moreover, neat Flamma® had no excitation peak in the range of 540 nm to 680 nm (Fig. 4a). After Flamma® conjugation, a strong excitation peak with a maximum at 618 nm was observed, which overlapped well with the upconversion emission bands of UCNPs, indicating energy transfer from UCNPs to Flamma® (Fig. 4a and c). This was confirmed by photoluminescence spectra; while UCNP@Ale-PEG nanoparticles showed no Flamma® emission after excitation at 618 nm, UCNP@Ale-PEG-Flamma® particles had ∼3 times higher luminescence at 816 nm than the free Flamma® (Fig. 4d). The limits and variations of the emission intensity of UCNP@Ale-PEG-Flamma® nanoparticles were demonstrated on aqueous dispersions of particles of different concentrations excited by a 980 nm laser at different power densities (Fig. S3 and S4†). The increased laser power clearly enhanced the luminescence intensity of Er^3+^ at 540 nm wavelength, while its red emission at 654 nm did not change at all laser powers (Fig. S3†). With decreasing particle concentration, there was minimal quenching of luminescence at the lowest particle concentration (Fig. S4†). It should also be noted that the intensity of the red emission was only slightly affected by the power density and depended on the particle concentration. This was attributed to the low Flamma® content in the dispersion and consequently low energy transfer.

Due to the low inherent chemical sensing capability of UCNPs, coupling with an external probe such as Flamma® can produce a biosensor enabling ratiometric imaging.^31^ UCNPs as donors can solve the existing problems of molecular Förster resonance energy transfer systems, such as photobleaching and quantitative analysis limitations.^32^ Conjugation of UCNPs with Flamma® dye thus represents an alternative approach to reduce the fluorescence background of conventional biosensors, which may enable imaging of a range of ions, toxins and biomolecular interactions.

### Comparing intraperitoneal *versus* intravenous delivery of UCNP@Ale-PEG-Flamma® nanoparticles to an orthotopically growing pancreatic cancer model

In animal experiments, Panc02 cells were orthotopically implanted into the surgically exposed pancreas of C57BL/6 mice to engraft an orthotopic model of pancreatic adenocarcinoma *in vivo*. This model mimics clinical human pancreatic tumors with similar desmoplastic stroma, tumor growth rate and metastatic profile. To study the biodistribution, UCNP@Ale-PEG-Flamma® nanoparticles were intraperitoneally or intravenously administered to mice 7 days after implantation of orthotopic tumor cells. Twenty-four hours later, the mice were sacrificed and the fluorescence signal from the dissected tissues was analyzed. Fluorescence extravital imaging revealed a large difference in the biodistribution of UCNP@Ale-PEG-Flamma® nanoparticles between two different systemic administration routes in the orthotopic pancreatic cancer model. It is important to note that this may not be consistent with subcutaneous models, which poorly mimic the clinical physiology of tumorous tissue.

**Fig. 6:**
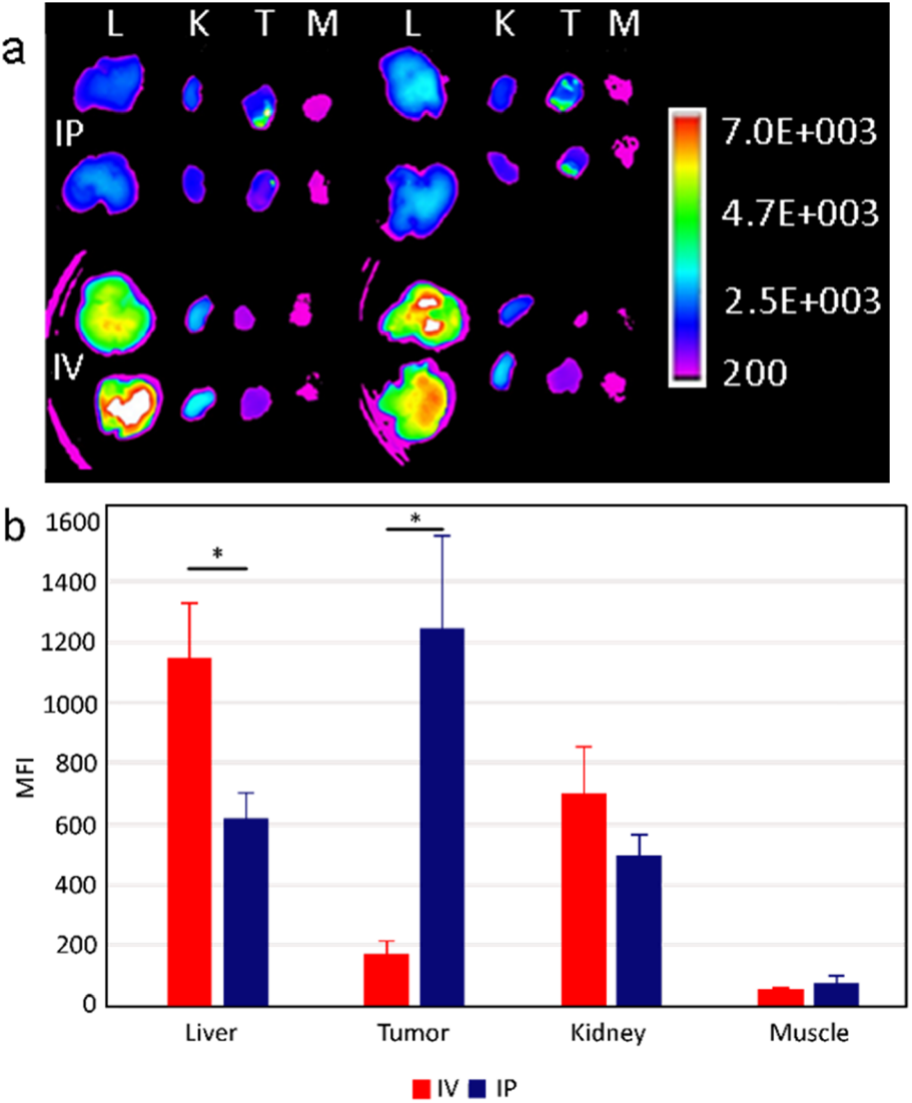
Fluorescence imaging of UCNP@Ale-PEG-Flamma® nanoparticles 24 h after intraperitoneal (IP) or intravenous (IV) administration in selected *ex vivo* tissues. (a) Top two rows represent accumulation of particles in tissues after IP application, bottom two rows represent IV administration. Tissue order from left to right: L – liver, K – kidney, T – tumor and M – skeletal muscle. (b) Mean fluorescence intensity (MFI) of particle accumulation in different tissues 24 h after IV and IP injection. *p < 0.05.

Intravenously administered UCNP@Ale-PEG-Flamma® particles accumulated strongly in the liver compared to intraperitoneal injection (Fig. 6a). The mean fluorescence intensity (MFI) was almost twice higher than that of the intraperitoneal route. The lower hepatic accumulation may be explained by the reduced clearance rate by the mononuclear phagocytic system. In contrast, significantly increased nanoparticle accumulation in orthotopic tumors was observed in animals administered by the intraperitoneal route. The measured MFI 24 h after injection was more than 6-fold higher than that of intravenous administration (Fig. 6b). In addition, a slightly increased fluorescence signal from the kidneys of mice was observed after intravenous administration. Although insignificant, this increase may be explained by direct exposure of intravenously administered UCNPs to renal excretion mechanisms, whereas intraperitoneal administration leads to lower (and delayed) blood levels (Fig. 6b). Moreover, no differences in particle accumulation were found in skeletal muscle, which was chosen as a negative control to the remaining three tissues where accumulation was anticipated. However, this was an expected finding as there is no reason for particle accumulation in skeletal muscle. The observed differences in nanoparticle distribution between intravenous and intraperitoneal administration may be attributed to distinct physiological barriers and clearance mechanisms involved. Intravenous administration exposes nanoparticles to immediate systemic circulation, leading to rapid clearance by organs such as the liver and kidneys. In contrast, intraperitoneal administration offers a localized delivery route that potentially bypasses some of the mechanisms of systemic clearance and allows for more targeted accumulation in peritoneal tumors. These differences in biodistribution underscore the importance of the administration route in achieving optimal therapeutic outcomes with nanoparticle-based drug delivery systems.

Oupicky and colleagues indeed observed the lack of mesothelium on both orthotopically growing tumors and even on spontaneous pancreatic tumors developed in a KPC mouse model.^16^ Interestingly, this was also confirmed in human pancreatic ductal adenocarcinoma patient samples, highlighting the potential translation of these findings into the clinical trials.

Nevertheless, despite these multiple supportive factors, the mechanism for the enhanced tumor accumulation efficiency of intraperitoneally administered UCNPs is still not fully understood, and a significant amount of UCNPs, for example, have entered the systemic circulation. Further optimization of the physicochemical and bioadhesive properties of UCNPs is then likely to enable improved intraperitoneal delivery and prolong their retention in the peritoneal cavity.

## Conclusions

In this study, we designed new Flamma®-conjugated PEG-alendronate-coated UCNPs. The particles were prepared by thermal coprecipitation of yttrium, ytterbium and erbium chlorides, coated with PEG-alendronate and conjugated with Flamma®. PEG-Ale coating provided excellent colloidal and chemical stability of UCNPs in water, DMEM, and ALF for at least 7 days, which is sufficient for most bioapplications. In general, chemical modification of surface properties and functionalization of UCNPs aims to improve the properties of drug delivery systems in precision medicine. Nanoparticle upconversion is then particularly beneficial in that it uses low-energy infrared light that penetrates relatively deep into living organs with very little damage to surrounding healthy tissue. The advantage of nanosized particles is that they improve drug delivery to specific target organs through active or passive targeting, thereby achieving high drug efficacy and safety. The small particle size enables good internalization into soft tumor tissues due to easy penetration across biological barriers. UCNPs also significantly improve traditional biological imaging of cells and tissues using fluorescence microscopy that just cannot be done otherwise. In our case, we investigated the biodistribution and pancreatic tumor accumulation of a water-soluble sulfo-NHS ester Flamma® fluorescent dye conjugated to UCNPs *via* PEG-Ale linkage. This novel combination of UCNPs and Flamma® has not been previously described and offers several advantages for monitoring drug biodistribution. Flamma® has the advantage of good reactivity with amino groups from deprotected PEG-Ale and superior fluorescent properties compared to commonly used dyes such as Cy7, whose hydrophobicity can negatively affect the results of biodistribution studies. The findings revealed a significant difference between commonly used intravenous and intraperitoneal delivery routes of UCNPs in an orthotopic mouse pancreatic cancer model. Intraperitoneal injection resulted in significantly higher nanoparticle accumulation in tumor tissue, with the mean fluorescence intensity (MFI) 24 h after injection being more than six times higher compared to the intravenous route. This is a major discovery for the development of novel drug delivery systems targeting tumor tissues, as enhanced tumor accumulation can potentially improve therapeutic efficacy and reduce systemic side effects. From a practical standpoint, intraperitoneal injection is easier to perform than intravenous administration, reducing technical difficulties and potentially improving the reproducibility of experimental results. In summary, our study demonstrated the superior pancreatic tumor-targeting capability of intraperitoneally administered UCNPs with conjugated Flamma® dye, highlighting the potential of this approach for enhanced pancreatic tumor imaging and drug delivery. The improved biodistribution profile observed with intraperitoneal administration could pave the way for more effective and less invasive pancreatic cancer therapies, making this a promising avenue for future research and clinical applications. Moreover, by combining upconversion luminescence and luminescence resonance energy transfer, new UCNP systems can be developed to enable the design of novel bioanalytical tools with improved functionality in optoelectronic applications.

## Data availability

The data supporting this article have been included as part of the ESI.†

## Author contributions

TV – investigation, VP – methodology, DV – formal analysis, OS – data curation, JP – visualization, MK – investigation, JB – formal analysis, DH – conceptualization and writing.

## Conflicts of interest

There are no conflicts to declare.

## Supplementary Material

NA-007-D4NA00764F-s001
