# Supplementary material for: Intraperitoneal versus intravenous administration of Flamma®-conjugated PEG-alendronate-coated upconversion nanoparticles in a mouse pancreatic cancer model
Source: Nanoscale Adv. 2024 Oct 25;7(1):144–54. doi: 10.1039/d4na00764f (PMC11575528; doi:10.1039/d4na00764f)
Supplement: NA-007-D4NA00764F-s001 [file NA-007-D4NA00764F-s001.pdf]

Supplementary Information (SI) for Nanoscale Advances.
This journal is © The Royal Society of Chemistry 2024

Supporting Information

Intraperitoneal *versus* intravenous administration of Flamma®-conjugated PEG-alendronate-coated upconversion nanoparticles in mice pancreatic cancer model

Taras Vasylyshyn[a], Vitalii Patsula[a], David Větvička[b], Oleksandr Shapoval[a], Jan Pankrác[c], Martina Kabešová[b], Jiří Beneš[b], Daniel Horák[a]

[a] Institute of Macromolecular Chemistry, Czech Academy of Sciences, Heyrovského nám. 2, 162 06 Prague 6, Czech Republic
[b] Institute of Biophysics and Informatics, First Faculty of Medicine, Charles University, Salmovská 1, 120 00 Prague 2, Czech Republic
[c] Center for Advanced Preclinical Imaging, First Faculty of Medicine, Charles University, Salmovská 3, 120 00 Prague 2, Czech Republic

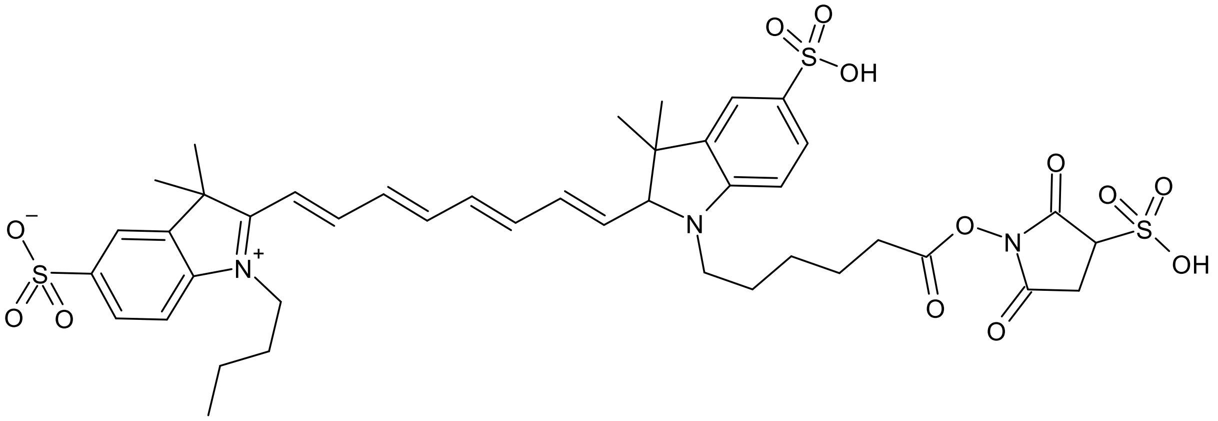

Figure S1. Formula of Flamma® 749 sulfo-NHS ester.

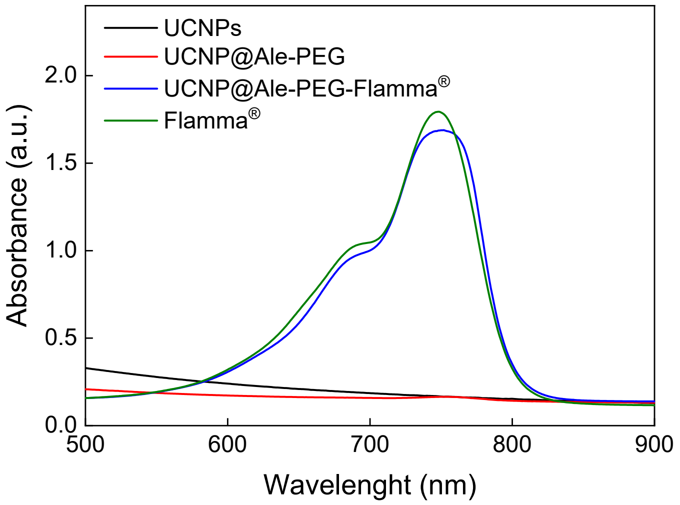

Figure S2. UV–Vis spectra of UCNPs, UCNP@Ale-PEG, UCNP@Ale-PEG-Flamma® and Flamma®.

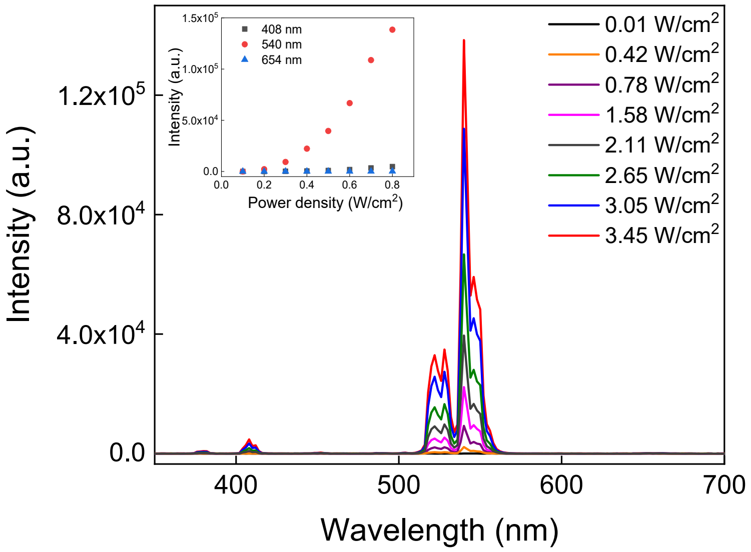

Figure S3. Upconversion photoluminescence emission spectra of aqueous UCNP@Ale-PEG-Flamma® dispersions (2 mg/ml) excited at 980 nm with different power densities. The inset shows the dependence of peak intensity on particle concentrations.

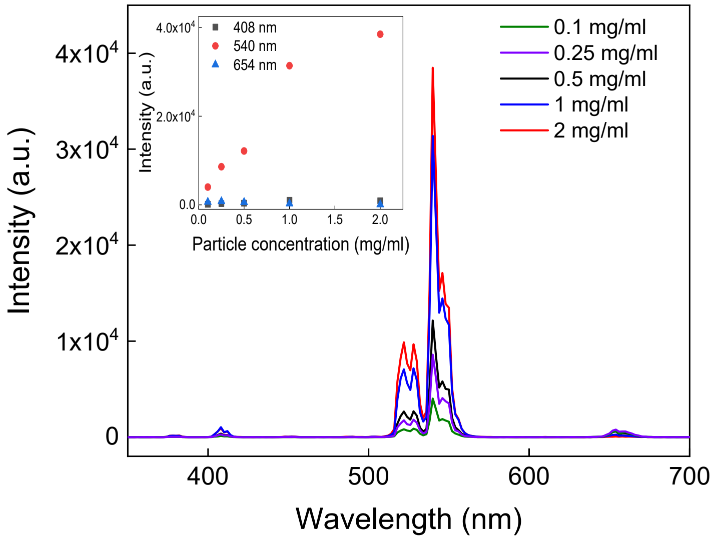

Figure S4. Upconversion photoluminescence emission spectra of aqueous UCNP@Ale-PEG-Flamma® dispersions at different concentrations; excitation at 980 nm, power density 2.11 W/cm^2. The inset shows the dependence of peak intensity on laser power density.
